# Association of kynurenine aminotransferase II gene C401T polymorphism with immune response in patients with meningitis

**DOI:** 10.1186/1471-2350-12-51

**Published:** 2011-04-07

**Authors:** Fladjule Rejane Soares de Souza, Fabrícia Lima Fontes, Thayse Azevedo da Silva, Leonam Gomes Coutinho, Stephen L Leib, Lucymara Fassarella Agnez-Lima

**Affiliations:** 1 Departamento de Biologia Celular e Genética, Universidade Federal do Rio Grande do Norte, Natal, Brazil; 2 Institute for Infectious Diseases, University of Bern, Friedbuehlstrasse 51, CH-3010 Bern, Switzerland

**Keywords:** Kynurenine Pathway, Polymorphism, Meningitis, Inflammatory response

## Abstract

****Background**:**

The kynurenine (KYN) pathway has been shown to be altered in several diseases which compromise the central nervous system (CNS) including infectious diseases such as bacterial meningitis (BM). The aim of this study was to assess single nucleotide polymorphisms (SNPs) in four genes of KYN pathway in patients with meningitis and their correlation with markers of immune response in BM.

**Methods:**

One hundred and one individuals were enrolled in this study to investigate SNPs in the following genes: indoleamine-2,3-dioxygenase (*IDO1 *gene), kynureninase (*KYNU *gene), kynurenine aminotransferase I (*CCBL1 *gene), and kynurenine aminotransferase II (*AADAT *gene). SNP analyses were performed by primer-introduced restriction analysis-PCR (PIRA-PCR) followed by RFLP. Cytokines were measured using multiplex bead assay while immunoglobulins (IG) by immunodiffusion plates and NF-kappaB and c-Jun by dot blot assay.

**Results:**

The variant allele of SNP *AADAT*+401C/T showed prevalent frequency in patients with BM. A significant decrease (*p *< 0.05) in TNF-α, IL-1β, IL-6, MIP-1αCCL3 and MIP-1β/CCL4 levels was observed in BM patients homozygous (TT) to the SNP *AADAT*+401C/T. Furthermore, a significant (*p *< 0.05) decrease in cell count was observed in cerebrospinal fluid (CSF) from patients with TT genotype. In addition, an increase in the IgG level in adults (*p *< 0.05) was observed. The variant allele for *KYNU*+715G/A was found with low frequency in the groups, and the SNPs in *IDO1*+434T/G, *KYNU*+693G/A, *CCBL1*+164T/C, and *AADAT*+650C/T had no frequency in this population.

****Conclusions**:**

This study is the first report of an association of SNP *AADAT*+401C/T with the host immune response to BM, suggesting that this SNP may affect the host ability in recruitment of leukocytes to the infection site. This finding may contribute to identifying potential targets for pharmacological intervention as adjuvant therapy for BM.

## Background

Bacterial meningitis (BM) is a severe infectious disease of the central nervous system (CNS) associated with acute inflammation that contributes to the development of subsequent brain damage. Despite the availability of effective antimicrobial therapy and intensive care, the outcome of meningitis remains associated with a high mortality. Moreover, brain and/or cochlear damage occur in up to 50% of the survivors [1]. An overactive immune response and the subsequent oxidative stress production, rather than the bacterial pathogen *per se*, are thought to be responsible for the neuronal damage, such as hearing loss and cognitive impairment [2].

Extensive research has been done in the last decades on the role of tryptophan (TRP) metabolism in the CNS under normal and pathological conditions. In recent years, a clear association has been made between tryptophan catabolism and inflammatory reactions in a vast array of disease states. Much of the focus of this research has centered on the kynurenine pathway of tryptophan degradation and the immune response [3,4].

Bacterial infections and lipopolysaccharide (LPS) application are strong inducers of indoleamine-2,3-dioxygenase (IDO), the enzyme responsible for converting tryptophan to kynurenine (KYN) in the brain [3,4]. The kynurenine pathway is activated by inflammatory mediators, e.g., free radicals and cytokines, which up-regulate *IDO1 *gene [5]. In sequence, KYN is converted to 3-hydroxykynurenine (3-HK) by kynurenine-3-hydroxylase (KMO). Both KYN and 3-HK can be oxidized by kynureninase (KYNU) to anthranilic acid (AA) or 3-hydroxyantrhanilic acid (3-HAA), respectively; or they can be transaminated by kynurenine aminotransferase (KAT) to kynurenic acid (KYNA) or xanthurenic acid (XA), respectively. Finally, 3-HAA can be oxidized to quinolinic acid (QUINA) by 3-hydroxyanthranilic acid oxidase (3-HAO) [3,4].

The rate of metabolism of TRP into the oxidative KYN pathway is controlled by IDO enzyme, which is induced, among other factors, by interleukin-1-beta (IL-1β), and tumor-necrosis factor alpha (TNF-α) [6,7]. These two pro-inflammatory cytokines are up-regulated in BM. TNF-α mediates many of the pathophysiological changes characteristic of BM, including blood-brain-barrier (BBB) breakdown, generation of the neutrophilic inflammation, increase in cerebral metabolism, oxygen consumption and cerebral blood flow [8,9]. On the other side, QUINA induces astrocytes to produce the pro-inflammatory chemokines monocyte chemoattractant protein (MCP-1/CCL2) and interleukin-8 (IL-8/CXCL8). These findings suggest that QUINA may be critical in the amplification of brain inflammation [10]. In contrast, KYNA was able to attenuate LPS-induced TNF-α secretion in a dose-dependent manner, acting as a ligand for the receptor for G protein-coupled receptor 35 (GPR35) [11].

Neurotoxic and neuroprotective activities have been attributed to different intermediary products of the KYN pathway. For example, QUINA acts as an agonist at N-methyl-D-aspartate (NMDA) receptors [12] and may cause neuronal excitotoxicity [13,14]. On the other hand, KYNA plays a protective role by acting as an antagonist of NMDA receptors [15,16]. Further, 3-HK and 3-HAA generate reactive oxygen species and, thus, induce neuronal damage [17,18].

Accumulation of neurotoxic intermediates of the KYN pathway was observed in the cortex and hippocampus of rats during the early and late phases of acute experimental bacterial meningitis [19]. Data suggesting that concentrations of KYN pathway metabolites are significantly altered in human patients with BM compared with controls have already been reported [20]. The KYN pathway is also induced in other diseases associated with inflammation-induced brain injury, such as Huntington's disease [21], schizophrenia [22], multiple sclerosis AIDS-dementia complex and cerebral malaria [23,24]. Association between the activation of the KYN pathway and inflammatory mediators has also been established and a negative feedback mechanism that downmodulates neuroinflammation in experimental models was proposed [for review see [4,25]].

In general, bacterial microorganisms that cause BM are oropharyngeal commensal and the disease development occurs from a previous infection, as sinusitis or otitis, with the blood-brain barrier breaching. Thus, the incidence of BM could be avoided by an efficient innate and acquired immune response. Concerning the involvement of KYN pathway with inflammatory response, the aim of this study was to investigate if single nucleotide polymorphisms (SNPs) in the KYN pathway genes could affect the immune response and be associated to BM. The identification of SNPs may be useful for characterization of populations susceptible to developing the disease, the infection severity and, consequently, the outcome. In the present study, we have attempted to study SNP in KYN pathway genes in meningitis patients in order to determine if any significant variation is associated with the disease. The analysis was based on primer introduced restriction analysis-PCR (PIRA-PCR) followed by RFLP. The analysis also associated the genotypes with the levels of cytokines, cell account, immunoglobulins, NF-kappaB, and c-Jun present in the cerebrospinal fluid (CSF) or plasma of the patients.

## Methods

### Patients and samples

Blood and CSF samples were obtained from 47 patients who were admitted at the Hospital Giselda Trigueiro (reference for Infectious Diseases in Natal-RN, Brazil) and had a positive diagnosis for BM. These diagnostics obtained from routine tests performed in the hospital were based on detection of the pathogen in the CSF by gram staining, bacterial culture, or antigen testing, and the presence of CSF pleocytosis. In total, 101 individuals (56 men and 45 women) were included in this study, with 28 individuals less than 18 years old, 69 adults between 19 and 60 years old, and 4 individuals aged over 60. Of the 47 patients with positive diagnosis for BM, 15 were diagnosed with *S*. *pneumonia*, 6 with *N. meningitides*, 7 with other pathogens, and 19 without specified etiology. The clinical parameters observed were cell count (2.887,6 ± 4.120,6 cells/mm^3^), glucose (35.9 ± 33.30 g/ml) and protein (178.1 ± 156.0 mg/d). Individuals in therapies or with others diseases (as AIDS) that affect the immune and inflammatory responses (e.g. the cytokines expression) were not included in the analysis, since these conditions might interfere in the results.

Aliquots of blood and CSF collected for the diagnosis of the disease were used in this work. Samples of CSF were collected just upon lumbar puncture and centrifuged at 720 g for 5 min. Supernatants were frozen and stored at -80°C before any further procedure. Blood samples were processed by centrifugation at 2,880 g for 3 min to separate plasma from pellet. Both were frozen and stored at -80°C. As control group, 54 blood samples were obtained from health volunteers and from patients attended at the Hospital Giselda Trigueiro who had the negative diagnosis for infectious disease. These patients were used as control since no infection was confirmed and all parameters were normal. CSF and blood were collected for diagnosis routine and all data about clinical parameters as glucose, pleocytosis, cytokines level were obtained from these patients. In health volunteers, CSF sample was not collected, since lumbar puncture is a very invasive method. This project was approved by the Brazilian Ethics Committee (CONEP, CAAE - 0052.1.051.000.05) and written consent was obtained from all patients involved. For children included in this study, the informed consent was obtained from their parents or guardians.

### Oligonucleotides

A survey of genes for KYN pathway enzymes was carried out using the NCBI database http://www.ncbi.nlm.nih.gov. The localization of protein conserved domains was obtained through the alignment of this region with the mRNA sequence using the program GeneWise, Expasy http://www.ebi.ac.uk/Wise2/index.html and the exons of interest were chosen by analysis of alignments of mRNA with the genomic DNA obtained with the tool Spidey from the NCBI database. SNPs were searched in functional domain regions of KYN pathway enzymes in the Human Genome Sequence (Table 1). Primers were designed for PIRA-PCR (Table 1) using Primer2 software [26].

**Table 1:** SNP data: primer sequences, annealing temperatures, PCR product sizes, and enzyme restrictions.

SNP	RS_ID	Alleles	Primer Sequence (5'→ 3')	Annealing Temp. (°C)	Product size (pb)	Restriction enzyme
*IDO1*+434T/G	rs4463407	T/G	F: TCAGGTCTTGCCAAGAACTA R: CAGTTTGCCAAGACACAGTC	53.5	101	MaeI
*KYNU*+693G/A	rs2304705	G/A	F: CGGACTTAACATTGAAGAAAGTATGC R: TTTGAGGAAAATGAAGAAAAAAATCA	55.0	115	HpyCH4V
*KYNU*+715G/A	rs6743085	G/A	F: ATATCCTCTATTCTTAAGGTTTCATC R: ATTTAGATCGGCAATATGAAAT	51.5	101	FokI
*CCBL1*+164T/C	rs17853193	T/C	F:CCAAGGCGTGACTTCAGGGC R:GGCACATGGGGAGAGTGTAGGACTAA	58.0	119	HaeIII
*AADAT*+650G/T	rs17852900	G/T	F:ATAGATCCATTTGTCCTTGACTGGAT R:ACATTTTTTGCTTTAGAATTCCAGAG	58.0	113	FokI
*AADAT*+401C/T	rs1480544	C/T	F:ACTATAGAAATCAATAACCCTAGAA R:GAAAACAAATTCTTATAGCCTG	50.0	111	MboII

### PIRA-PCR and Genotyping

The genomic DNA samples were extracted from blood samples (2-5 ml) using a salting out procedure [27]. PCR amplification was carried out in a total volume of 25 μl containing 2.5 μl of 10×PCR buffer, 200 ng of genomic DNA, 0.1 mM of dATP, dGTP, dCTP and dTTP (Invitrogen), 20 pmol of each primer, 1.5 mol/l MgCl2, and 1 U Taq polymerase (Invitrogen). Amplifications were performed in a DNA thermal cycler (Eppendorf) for 35 cycles. The reaction conditions were: pre-denaturation at 94°C for 5 min, followed by 35 cycles of denaturation at 94°C for 45 s, annealing shown in Table 1 for 45 s, and extension at 72°C for 1 min. The sequences of primers and the lengths of the PCR products analyzed are also shown in Table 1. The primers contain a single-base mismatch leading to the production of PCR products containing a restriction site. Prior to digestion, samples were electrophoresed on 2% agarose gels containing ethidium bromide to verify amplification. An aliquot of each PCR product was digested with the respective enzyme (Table 1) in the buffer supplied by the manufacturer. The product of digestion was visualized in polyacrylamide gels 8% stained with silver [28]. The analysis of genotyping was blinded and all samples were replicated.

### Measurement of chemokines and cytokines

Chemokine and cytokine levels in CSF were measured by a Bio-Plex 200 suspension array system (Bio-Rad) using microsphere-based multiplex assays. A human cytokine Lincoplex Kit (HCYTO-60k, Lincoplex^®^, Linco Research Inc.) was used in this assay. This kit allows the detection of TNF-α, IL-6, IL-1β, INF-γ, IL-10, IL-1Ra, MIP-1α/CCL3 and MIP-1β/CCL4, MCP-1/CCL2, G-CSF, IL-8/CXCL8, and GM-CSF. The assay was performed as previously described by Gehre et al., 2008 [29]. Cytokine concentrations were calculated using a standard curve derived from a recombinant standard cytokine by Bio-Plex Manager software. Samples were assessed undiluted and serially diluted to allow quantification of cytokines over a broad range (3.2-10,000 pg/ml).

### Measurements of immunoglobulins

The total levels of IgG and IgA were measured from the patients' plasma, using quantitative radial immunodiffusion plates (Diffu-plate, Biocientífica SA), according to the manufacturer's instructions. Due to the low quantity of biological material, it was not possible to make the determination in all the patients.

### Dot Blot assay to NF-kappaB and c-Jun

Dot blot assay was performed using an adapted protocol based on Towbin et al. (1979) [30]. Briefly, proteins were obtained from aliquots containing 10 μl of CSF that were previously centrifuged for 15,000 g, 4°C, for 5 min. Samples were transferred to an activated PVDF membrane (Qbiogene) and nonspecific binding was avoided by incubation in a blocking buffer (5% skimmed milk (non-fat) diluted in TBST) for one hour. Membranes were then probed with primary monoclonal antibodies against NF-kappaB (Santa Cruz Biotechnology) and c-Jun (R&D systems) diluted in blocking buffer. After washing, blots were incubated with secondary antibodies conjugated to horseradish-peroxidase (Santa Cruz Biotechnology) diluted in blocking solution. Protein-antibody reactions were detected through reaction with 0.5% DAB (Across Organics), AcNH_4 _(50 mM, pH 5.0), and 0.06% H_2_O_2 _(Merck). Detection of β-actin antibody was used as control of endogenous protein expression. For the quantification of protein levels, dots were photographed and images were analyzed with the *ImageJ *program (NIH, public domain). Estimation of the relative NF-kappaB and c-Jun levels was obtained by the ratio in relation to the β-actin level.

### Statistical analysis

Allelic frequencies were determined by direct count of the alleles. Genotypic distributions were examined for significant departure from Hardy-Weinberg equilibrium by classical method of *X*^2^-test (two-tailed) using Helix SVS program. The significance among differences in allelic and genotypic frequencies observed between all analyzed groups was evaluated by *X*^2^-test (two-tailed) using WINPEPI program. Differences in levels of inflammatory markers were analyzed by non-parametric t-test Mann-Whitney U (two-tailed) and spearman correlation test was carried out with *GraphPad-Prism5 *software. The findings were considered significant when *P *< 0.05.

## Results

The results obtained for the SNP *AADAT*+401C/T showed that among the 101 analyzed individuals the allelic frequencies were 0.59 and 0.40 for the alleles C and T respectively. Moreover, the analysis of the sub-groups shows differences in the allelic and genotypic frequencies. All groups are in Hardy-Weinberg equilibrium. In the BM patients, the frequency of T allele was 0.51, while for the control patients the frequency was 0.3 (*P *= 0.004, OR = 2.49). An increase for the TT genotype was also observed in the BM group, suggesting the association of this SNP with the disease (Table 2).

**Table 2:** Genotypic and allelic frequencies of SNPs *AADAT*+401C/T and *KYNU+*715G/A.

*Genotype AADAT*+401C/T	*BM Cases 47 (%)*	*Controls 54 (%)*	*OR (95% CI)*^a^	*P-value*^b^	*CC vs TT*^d^
CC	11 (23)	28 (52)	0.275 (0.150 to 0.507)	<0.0001^c^	*P *= 0.021 ^c^
CT	24 (51)	19 (35)	1.933 (1.095 to 3.411)	0.0223^c^	OR = 0.23 (0.07 to 0.72)
TT	12 (25)	7 (13)	2.231 (1.066 to 4.667)	0.0305^c^	
*Frequency of T allele*	0.51	0.30	2.429 (1.359 to 4.339)	0.0038^c^	

***Genotype KYNU*+715G/A**	***BM Cases 46 (%)***	***Controls 52 (%)***	***OR (95% CI)*^a^**	***P-value*^b^**	***GG vs AA*^d^**

GG	41 (89)	43 (82)	1.776 (0.7916 to 3.985)	0.1598	*P *= 0.38
GA	2 (4)	4 (7)	0.5536 (0.1568 to 1.954)	0.3521	OR = 1.91(0.45 to 8.00)
AA	3 (7)	6 (11)	0.6090 (0.2260 to 1.641)	0.3230	
*Frequency of A allele*	0.08	0.15	0.4928 (0.1988 to 1.221)	0.1208	

^a ^ORs and probability values at 95% CI for disease status are shown.

^b ^Values were calculated with the X^2^-test (two-tailed) comparing controls and case patients.

^c ^Significant *P *value (*P *< 0.05).

^d ^Analysis of distribution of the contrasting genotypes. Values calculated with X^2^-test (two-tailed)

In this population (101 individuals), the variant allele frequency observed for the SNP *KYNU*+715G/A was 0.0104. No significant differences were observed between BM patients and control group (Table 2).

The variant alleles for SNPs *KYNU*+693G/A, *CCBL1*+164T/C, *AADAT*+650G/T and *IDO1*+434T/G were not found in this population. The frequency of these SNPs should be very low and it was not possible to find the polymorphic allele among the 101 analyzed samples. Thus, this analysis does not suggest association of SNPs *KYNU*+715G/A, *KYNU*+693G/A, *CCBL1*+164T/C, *AADAT*+650G/T, and *IDO1*+434T/G with BM. However, the SNP *AADAT*+401C/T may be associated with the disease, since allelic and genotypic frequencies show statistical significance between BM patients and control group.

**Figure 1:**
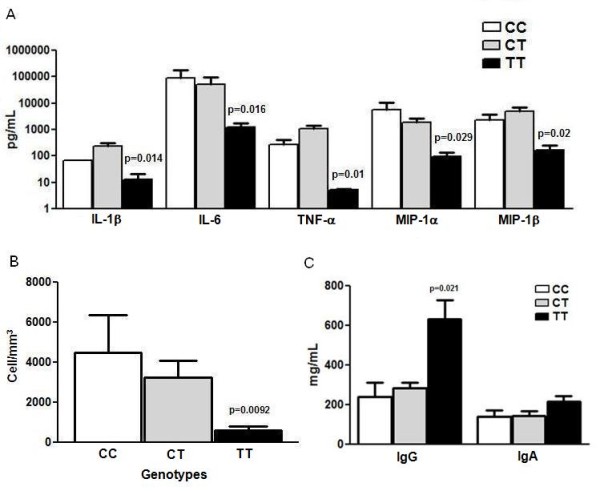
**Markers of immune response associated to SNP *AADAT*+401C/T**. a) Cytokine and chemokine concentration in CSF samples of BM patients in relation to the genotype for SNP *AADAT*+401C/T; b) Cell counts associated to *AADAT*+401C/T genotype; c) IgG and IgA concentration in plasma samples from adults patients in relation to the genotype for SNP *AADAT*+401C/T. All *P *values are related to TT compared to CC genotype.

Concentrations of cytokines and chemokines were measured in CSF samples of BM patients. A significant (*p *< 0.05) decrease in levels of TNF-α, IL-1β, IL-6, MIP-1α/CCL3 and MIP-1β/CCL4 was observed in patients homozygous (TT) to the SNP *AADAT*+401C/T (Figure 1a). No significant differences were observed for INF-γ, IL-10, IL-1Ra, MCP-1/CCL2, G-CSF, IL-8/CXCL8 and GM-CSF levels (data not shown) and in NF-kappaB and c-Jun expression (data not shown). A significant reduction in cell count was observed in patients with TT genotype (Figure 1b). The correlation analyses (Table 3) showed that the cell count has significant correlation with TNF-α, IL-1β, IL-6, MIP-1αCCL3 and MIP-1β/CCL4 in all patients. However, the analyses in relation to genotype showed differences between CC or TT genotypes. Significant correlation between cell count and TNF-α, IL-6, MIP-1αCCL3 and MIP-1β/CCL4 was observed in patients with TT genotype, while for patients with CC genotype significant correlation was only observed between cell count and IL-6.

**Table 3:** Correlation between cell count and cytokines and chemokines level in BM patients

All patients	TNF-α	IL-6	IL-1β	MIP-1β	MIP-1α
Spearman r	0.6181	0.5308	0.6218	0.5295	0.4287
*P *value	<0.0001^a^	0.0002 ^a^	<0.0001^a^	0.0002^a^	0.003^a^

**CC genotype**	**TNF-α**	**IL-6**	**IL-1β**	**MIP-1β**	**MIP-1α**

Spearman r	0.2848	0.8283	0.2439	0.3404	0.2364
*P *value	0.4250	0.0031^a^	0.4971	0.3358	0.5109

**TT genotype**	**TNF-α**	**IL-6**	**IL-1β**	**MIP-1β**	**MIP-1α**

Spearman r	0.8500	0.8034	0.6390	0.9500	0.7833
*P *value	0.0061 ^a^	0.0138 ^a^	0.666	0.0004 ^a^	0.0172 ^a^

- Correlation analysis done using Spearman test two-tailed (cell count vs cytokine or chemokine level)

^a ^Significant *P *value (*P *< 0.05).

Due to differences in acquired immune response in children (<18 years old) and adults (≥ 18 years old), each group was subjected to a distinct analysis of IgG level. Significant increase in the IgG level was observed in adult patients with TT genotype (Figure 1c). The number of children in our sample was not enough for the statistical analysis of IgG level, however the same tendency was observed (data not shown).

## Discussion

In the present study, the association of SNPs in key enzymes of the KYN pathway with bacterial meningitis was investigated in Brazilian patients. The TT genotype for SNP *AADAT*+401C/T show a high frequency in patients with BM compared with the control group (Table 2). This result shows indicative that the variant allele *AADAT*+401C/T may play a relevant role in inflammatory regulation during the development of the disease. However, the number of patients included in this work is limited and further studies are required in larger cohorts.

The enzyme KATII (product of *AADAT *gene) is responsible for the formation of KYNA found in glial and neuronal cells [31]. The SNP *AADAT*+401C/T is located in a putative exonic splicing silencers (ESSs), and mutations in these regions may result in quantitative changes in the production canonical mRNAs and peptide production that are likely to contribute significantly to inter-individual phenotypic variability [32].

Bacterial microorganisms that cause BM are usually oropharyngeal commensal in a fraction of the population. The disease develops when the pathogens gain access to the CSF space either via the blood stream in bacteremia or from an adjacent infection e.g from sinusitis. Thus, the incidence of BM could be lowered by an efficient immune defense [33]. A critical role of cytokines and chemokines has been carefully established in models of bacterial meningitis. Following the injection of LPS from gram-negative meningeal pathogens as *Neisseria meningitides *or cell-wall components from pneumococci, the rapid increase of proinflammatory cytokines (as TNF-α, IL-1β and IL-6) can be documented in CSF, which is followed by the recruitment of granulocytes and increased CSF protein concentrations [34-36].

High levels of TNF-α, IL-6 and IL-1β are characteristic of meningitis processes and may be associated with disease severity and the occurrence of sequelae [37]. Numerous studies have demonstrated that these cytokines are important to activation of chemokines (such as MIP-1α/CCL3 and MIP-1β/CCL4) involved with the leukocyte recruitment to the infection site for effective pathogen eradication [38]. In several models of infection, the deficiency in these cytokines and chemokines was associated with the decrease in the clearance of pathogens, development of invasive disease, and high mortality [39-41]. Furthermore, the correlation of chemokines with cell count in CSF during BM have also been reported [38,42]. Low levels of IL-1β, TNF-α and IL-6 in nasopharyngeal secretion were observed in children with recurrent episodes of acute otitis media [43], an important cause of meningitis.

Our results suggest that the SNP *AADAT*+401C/T may affect the expression of markers of the immune response characteristic in meningitis. We observed a significant reduction (*P *< 0.05) in the level of TNF-α, IL-1β, IL-6, MIP-1α/CCL3 and MIP-1β/CCL4 in BM patients who had the TT genotype (Figure 1a) and also a reduction in cell count (Figure 1b) with the highest correlation with the cytokines and chemokines (Table 3), suggesting a reduced ability in leukocyte recruitment in these patients.

The KYN pathway has been described as involved in the regulation of inflammatory response. However the mechanisms of regulation are not well understood [3,4,25]. NF-kappaB and Ap-1 (Fos/Jun) play a crucial role during induction of inflammatory response to BM. These transcriptional factors are involved in very complex cascades of events that lead to induction of cytokines, chemokines and adhesion molecules [37,44]. Differences in expression of NF-kappaB and c-Jun in relation to genotypes were not observed in our work. These data suggest that the effect of the SNP *AADAT*+401C/T may be downstream of the activation of NF-kappaB during inflammatory response. Recently, Ogasawara et al. (2009) [45] showed that the activation of NF-kappaB through the PI3K pathways is required for the IDO expression induced by hemoglobin in bone marrow-derived myeloid dendritic cells. This finding suggests that activation of NF-kappaB precedes the KYN pathway activation.

KYNA was identified as a ligand for the receptor for GPR35, proposing a novel mechanism by which KYNA may regulate peripheral cellular responses through activation of GPR35. KYNA was able to attenuate LPS-induced TNF-α secretion in a dose-dependent manner. The predominant expression of GPR35 in immune cells and the elevation of KYNA levels during inflammation suggested that this receptor-ligand complex may play important roles in immunological regulation. Because the tryptophan metabolic pathway is activated by pro-inflammatory stimuli, the anti-inflammatory effect of KYNA provides an interesting feedback mechanism in modulating immune responses [11].

Positive correlation between CSF KYNA levels with age and IgG and β_2_-microglobulin levels was observed in human subjects without neurological disease [46]. These authors propose that the enhancement of CSF IgG and β_2_-microglobulin levels would suggest an activation of the immune system during aging. Moreover, the increased KYNA metabolism would be involved in the hypofunction of the glutamatergic and/or nicotinic cholinergic neurotransmission in the CNS aging. However, the relationship between KYNA changes and activation of IgG and β_2_-microglobulin levels with aging is not yet clear. In our work, we also observed an association of TT genotype (SNP *AADAT*+401C/T) with an increase of IgG level. This evidence indicates that the KYNA level affects the antibody production by an unknown mechanism.

Although the population studied in this work has small size, our sampling represents the epidemiological and clinical spectrum of BM in our region. The low frequency of meningitis cases and the poor public health system in Natal (Brazil) did not allow the more accurate stratification of the patients and the examination of the influence of level-altered chemokines and cytokines in severity or sequelae in patients, pointing the need to extend the analysis done in this work for a large numbers of patients.

## Conclusions

Our data suggest that among the allele variants studied in this work the SNP *AADAT*+401C/T may be associated with meningitis. The allele variants for *KYNU*+715G/A, *KYNU*+693G/A, *CCBL1*+164T/C, *AADAT*+650G/T, and *IDO1*+434T/G had a lower frequency and probably are not associated with meningitis. In this research, we obtained indicatives that the presence of SNP in the *AADAT*+401C/T affects the expression of markers of the immune response to meningitis. However, these evidences need to be confirmed in further studies with larger cohorts. A better understanding of this correlation and the mechanisms involved is essential to identify potential targets for pharmacological intervention as an adjuvant therapy in BM as well as the possibility of stratification for risk assessment, genetic predisposition, diagnosis, and prognosis.

## Competing interests

The authors declare that they have no competing interests.

## Authors' contributions

LFAL and SLL conceived and coordinated the study. FRSS and FLF carried out the genotyping experiments and conducted the statistical analysis. LFA, FRSS and FLF drafted the manuscript. LGC measured chemokines and cytokines. TAS performed assay to immunoglobulins, NF-kappaB, and c-Jun. FRSS, TAS, and LGC collaborated in collection of the samples. All authors read and approved the final version.

## Pre-publication history

The pre-publication history for this paper can be accessed here:

http://www.biomedcentral.com/1471-2350/12/51/prepub

## References

[B1] Bedford H de Louvois J Halket S Peckham C Hurley R Harvey D Meningitis in infancy in England and Wales: follow up at age 5 years Bmj 2001 323 533 536 10.1136/bmj.323.7312.533 11546697 PMC48156

[B2] Pfister HW Scheld WM Brain injury in bacterial meningitis: therapeutic implications Curr Opin Neurol 1997 10 254 259 10.1097/00019052-199706000-00015 9229135

[B3] Stone TW Kynurenines in the CNS: from endogenous obscurity to therapeutic importance Prog Neurobiol 2001 64 185 218 10.1016/S0301-0082(00)00032-0 11240212

[B4] Moffett JR Namboodiri MA Tryptophan and the immune response Immunol Cell Biol 2003 81 247 265 10.1046/j.1440-1711.2003.t01-1-01177.x 12848846

[B5] King NJ Thomas SR Molecules in focus: indoleamine 2,3-dioxygenase Int J Biochem Cell Biol 2007 39 2167 2172 10.1016/j.biocel.2007.01.004 17320464

[B6] O'Connor JC André C Wang Y Lawson MA Szegedi SS Lestage J Castanon N Kelley KW Dantzer R Interferon-gamma and tumor necrosis factor-alpha mediate the upregulation of indoleamine 2,3-dioxygenase and the induction of depressive-like behavior in mice in response to bacillus Calmette-Guerin J Neurosci 2009 29 4200 4209 19339614 10.1523/JNEUROSCI.5032-08.2009 PMC2835569

[B7] Nisapakultorn K Makrudthong J Sa-Ard-Iam N Rerkyen P Mahanonda R Takikawa O Indoleamine 2,3-dioxygenase expression and regulation in chronic periodontitis J Periodontol 2009 80 114 121 10.1902/jop.2009.080315 19228097

[B8] Leib SL Clements JM Lindberg RL Heimgartner C Loeffler JM Pfister LA Täuber MG Leppert D Inhibition of matrix metalloproteinases and tumour necrosis factor alpha converting enzyme as adjuvant therapy in pneumococcal meningitis Brain 2001 124 1734 1742 10.1093/brain/124.9.1734 11522576

[B9] Waage A Halstensen A Shalaby R Brandtzaeg P Kierulf P Espevik T Local production of tumor necrosis factor alpha, interleukin 1, and interleukin 6 in meningococcal meningitis. Relation to the inflammatory response J Exp Med 1989 170 1859 1867 10.1084/jem.170.6.1859 2584928 PMC2189530

[B10] Guillemin GJ Croitoru-Lamoury J Dormont D Armati PJ Brew BJ Quinolinic acid upregulates chemokine production and chemokine receptor expression in astrocytes Glia 2003 41 371 381 10.1002/glia.10175 12555204

[B11] Wang J Simonavicius N Wu X Swaminath G Reagan J Tian H Ling L Kynurenic acid as a ligand for orphan G protein-coupled receptor GPR35 J Biol Chem 2006 281 22021 22028 10.1074/jbc.M603503200 16754668

[B12] Stone TW Perkins MN Quinolinic acid: a potent endogenous excitant at amino acid receptors in CNS Eur J Pharmacol 1981 72 411 412 10.1016/0014-2999(81)90587-2 6268428

[B13] Stone TW Kynurenines in the CNS: from endogenous obscurity to therapeutic importance Prog Neurobiol 2001 64 185 218 10.1016/S0301-0082(00)00032-0 11240212

[B14] Schwarcz R Whetsell WO Mangano RM Quinolinic acid: an endogenous metabolite that produces axon-sparing lesions in rat brain Science 1983 219 316 318 10.1126/science.6849138 6849138

[B15] Stone TW Darlington LG Endogenous kynurenines as targets for drug discovery and development Nat Rev Drug Discov 2002 1 609 620 10.1038/nrd870 12402501

[B16] Perkins MN Stone TW An iontophoretic investigation of the actions of convulsant kynurenines and their interaction with the endogenous excitant quinolinic acid Brain Res 1982 247 184 187 10.1016/0006-8993(82)91048-4 6215086

[B17] Eastman CL Guilarte TR Cytotoxicity of 3-hydroxykynurenine in a neuronal hybrid cell line Brain Res 1989 495 225 231 10.1016/0006-8993(89)90216-3 2765927

[B18] Okuda S Nishiyama N Saito H Katsuki H 3-Hydroxykynurenine, an endogenous oxidative stress generator, causes neuronal cell death with apoptotic features and region selectivity J Neurochem 1998 70 299 307 10.1046/j.1471-4159.1998.70010299.x 9422375

[B19] Bellac CL Coimbra RS Christen S Leib SL Pneumococcal meningitis causes accumulation of neurotoxic kynurenine metabolites in brain regions prone to injury Neurobiol Dis 2006 24 395 402 10.1016/j.nbd.2006.07.014 16956766

[B20] Coutinho LG Bellac C Grandgirard D Wittwer M Coimbra RS Christen S Agnez-Lima LF Marinho LA Leib SL Assessment of tryptophan metabolism and cytokine profile in cerebrospinal fluid samples from patients with bacterial meningitis Int J Antimicrob Agents 2007 21 S203 10.1016/S0924-8579(07)70647-7

[B21] Guidetti P Reddy PH Tagle DA Schwarcz R Early kynurenergic impairment in Huntington's disease and in a transgenic animal model Neurosci Lett 2000 283 233 235 10.1016/S0304-3940(00)00956-3 10754231

[B22] Schwarcz R Rassoulpour A Wu HQ Medoff D Tamminga CA Roberts RC Increased cortical kynurenate content in schizophrenia Biol Psychiatry 2001 50 521 530 10.1016/S0006-3223(01)01078-2 11600105

[B23] Nemeth H Toldi J Vecsei L Role of kynurenines in the central and peripheral nervous systems Curr Neurovasc Res 2005 2 249 260 10.2174/1567202054368326 16181118

[B24] Stone TW Mackay GM Forrest CM Clark CJ Darlington LG Tryptophan metabolites and brain disorders Clin Chem Lab Med 2003 41 852 859 10.1515/CCLM.2003.129 12940508

[B25] Kwidzinski E Bechmann I IDO expression in the brain: a double-edged sword J Mol Med 2007 85 1351 1359 10.1007/s00109-007-0229-7 17594069

[B26] Ke X Collins A Ye S PIRA PCR designer for restriction analysis of single nucleotide polymorphisms Bioinformatics 2001 17 838 839 10.1093/bioinformatics/17.9.838 11590100

[B27] Miller SA Dykes DD Polesky HF A simple salting out procedure for extracting DNA from human nucleated cells Nucleic Acids Res 1988 16 1215 10.1093/nar/16.3.1215 3344216 PMC334765

[B28] Sanguinetti CJ Dias Neto E Simpson AJ Rapid silver staining and recovery of PCR products separated on polyacrylamide gels Biotechniques 1994 17 914 921 7840973

[B29] Gehre F Leib SL Grandgirard D Kummer J Buhlmann A Simon F Gaumann R Kharat AS Tauber MG Tomasz A Essential role of choline for pneumococcal virulence in an experimental model of meningitis J Intern Med 2008 264 143 154 10.1111/j.1365-2796.2008.01930.x 18331292

[B30] Towbin H Staehelin T Gordon J Electrophoretic transfer of proteins from polyacrylamide gels to nitrocellulose sheets: procedure and some applications Proc Natl Acad Sci USA 1979 76 4350 4354 10.1073/pnas.76.9.4350 388439 PMC411572

[B31] Roberts RC Du F McCarthy KE Okuno E Schwarcz R Immunocytochemical localization of kynurenine aminotransferase in the rat striatum: a light and electron microscopic study J Comp Neurol 1992 326 82 90 10.1002/cne.903260107 1479071

[B32] Kralovicova J Vorechovsky I Global control of aberrant splice-site activation by auxiliary splicing sequences: evidence for a gradient in exon and intron definition Nucleic Acids Res 2007 35 6399 6413 10.1093/nar/gkm680 17881373 PMC2095810

[B33] Smirnova I Mann N Dols A Derkx HH Hibberd ML Levin M Beutler B Assay of locus-specific genetic load implicates rare Toll-like receptor 4 mutations in meningococcal susceptibility Proc Natl Acad Sci USA 2003 100 6075 80 10.1073/pnas.1031605100 12730365 PMC156328

[B34] Waage A Halstensen A Shalaby R Brandtz P Kierule P Espevik T Local production of tumor necrosis factor a, interleukin 1, and interleukin-6 in meningococcal meningitis J Exp Med 1989 170 1859 1867 10.1084/jem.170.6.1859 2584928 PMC2189530

[B35] Vermont CL de Groot R Hazelzet JA Bench-to-bedside review: genetic influences on meningococcal disease Crit Care 2002 6 60 65 10.1186/cc1454 11940267 PMC137398

[B36] Hirst RA Kadioglu A O'callaghan C Andrew PW The role of pneumolysin in pneumococcal pneumonia and meningitis Clin Exp Immunol 2004 138 195 201 10.1111/j.1365-2249.2004.02611.x 15498026 PMC1809205

[B37] Koedel U Scheld WM Pfister HW Pathogenesis and pathophysiology of pneumococcal meningitis Lancet Infect Dis 2002 2 721 736 10.1016/S1473-3099(02)00450-4 12467688

[B38] Zwijnenburg PJ van der Poll T Roord JJ van Furth AM Chemotactic factors in cerebrospinal fluid during bacterial meningitis Infect Immun 2006 74 1445 1451 10.1128/IAI.74.3.1445-1451.2006 16495514 PMC1418618

[B39] Lindell DM Standiford TJ Mancuso P Leshen ZJ Huffnagle GB Macrophage inflammatory protein 1alpha/CCL3 is required for clearance of an acute Klebsiella pneumoniae pulmonary infection Infect Immun 2001 69 6364 6369 10.1128/IAI.69.10.6364-6369.2001 11553580 PMC98771

[B40] MacLennan IC Germinal centers Annu Rev Immunol 1994 12 117 139 10.1146/annurev.iy.12.040194.001001 8011279

[B41] Janoff EN Rubins JB Invasive pneumococcal disease in the immunocompromised host Microb Drug Resis 1997 3 215 232 10.1089/mdr.1997.3.215 9270991

[B42] Inaba Y Ishiguro A Shimbo T The production of macrophage inflammatory protein-1alpha in the cerebrospinal fluid at the initial stage of meningitis in children Pediatr Res 1997 42 788 793 10.1203/00006450-199712000-00012 9396559

[B43] Lindberg K Rynnel-Dagoo B Sundqvist KG Cytokines in nasopharyngeal secretions; evidence for defective IL-1beta production in children with recurrent episodes of acute otitis media Clin Exp Immunol 1994 97 396 402 10.1111/j.1365-2249.1994.tb06101.x 8082294 PMC1534847

[B44] Vallabhapurapu S Karin M Regulation and function of NF-kappaB transcription factors in the immune system Annu Rev Immunol 2009 27 693 733 10.1146/annurev.immunol.021908.132641 19302050

[B45] Ogasawara N Oguro T Sakabe T Matsushima M Takikawa O Isobe K Nagase F Hemoglobin induces the expression of indoleamine 2,3-dioxygenase in dendritic cells through the activation of PI3K, PKC, and NF-kappaB and the generation of reactive oxygen species J Cell Biochem 2009 108 716 725 10.1002/jcb.22308 19693771

[B46] Kepplinger B Baran H Kainz A Ferraz-Leite H Newcombe J Kalina P Age-related increase of kynurenic acid in human cerebrospinal fluid - IgG and beta2-microglobulin changes Neuro-Signals 2005 14 126 135 10.1159/000086295 16088227

